# Multiple Basal Cell Carcinomas in Immunocompetent Patients

**DOI:** 10.3390/cancers14133211

**Published:** 2022-06-30

**Authors:** Paola Savoia, Federica Veronese, Lara Camillo, Vanessa Tarantino, Ottavio Cremona, Elisa Zavattaro

**Affiliations:** 1 Department of Health Sciences, University of Eastern Piedmont, Via Solaroli 17, 28100 Novara, Italy; lara.camillo@med.uniupo.it; 2 Azienda Ospedaliera Universitaria Maggiore della Carità, c.so Mazzini 18, 28100 Novara, Italy; federica.veronese@med.uniupo.it (F.V.); vanessa.tarantino@maggioreosp.novara.it (V.T.); elisa.zavattaro@med.uniupo.it (E.Z.); 3 Vita-Salute San Raffaele University, Via Olgettina 58, 20132 Milan, Italy; cremona.ottavio@hsr.it; 4 Department of Translational Medicine, University of Eastern Piedmont, Via Solaroli 17, 28100 Novara, Italy

**Keywords:** non-melanoma skin cancer, basal cell carcinoma, risk factors, UV-exposure, hedgehog pathway, mutational status

## Abstract

**Simple Summary:**

It is widely known that long-term treatment with immunosuppressive drugs represents a risk factor for the onset of malignancies, including multiple basal cell carcinomas. However, multiple basal carcinomas are ao found in the general population, and even in the absence of specific predisposing genetic mutations. This paper aims, through the retrospective evaluation of all patients diagnosed and surgically treated for basal cell carcinomas during 5 years at our Dermatological Division, to identify the characteristics of these subjects and any possible risk factors, useful for outlining specific surveillance programs. In our experience, multiple carcinomas were identified in over 24% of the subjects analyzed, with several lesions removed, ranging from 2 to 11, confirming the relevance of this phenomenon.

**Abstract:**

Background: The onset of multiple BCCs is a relatively common condition, not only among patients undergoing chronic treatment with immunosuppressant drugs, but also in the general population, although specific risk factors for immunocompetent patients have not been identified. A putative role of somatic mutations in the hedgehog pathway should be considered. Methods: This study is a retrospective observation of all patients diagnosed and surgically treated for BCCs during 5 years at our Dermatological Division. For these patients, we evaluated clinical and histopathological characteristics and data about possible risk factors for BCC. Results: Five-hundred and six patients affected by multiple BCCs, accounting for the 24.2% of the entire sample, have been identified. In these patients, the total number of BCCs was 1516, ranging from 2 to 11. Subjects affected by multiple BCCs were more frequently males, with an older age at diagnosis; multiple BCCs developed mainly on the trunk and were often represented by a nodular histotype. The multivariate analysis highlighted that male gender, older age, nodular BCC, or face involvement at the first diagnosis are risk factors for the development of multiple BCCs. Conclusions: The frequency of multiple BCCs even among the non-immunocompromised population underlines the need to subject patients to a close surveillance program, to allow early diagnosis and treatment of additional cancers.

## 1. Introduction

Basal cell carcinoma (BCC) is the most common human tumor; its incidence progressively increases, with important implications for the treatment needs of affected patients and healthcare costs [1]. The carcinogenic effects of UV radiation have a critical role in the pathogenesis of this cancer, and most BCCs develop in the head and neck region or other chronically photo-exposed areas [2]. However, 10–15% of BCCs arise on mostly covered anatomical sites [3], and the causes underlying this phenomenon have yet to be fully understood.

It is well known that long-term use of immunosuppressive drugs may cause the onset of multiple BCCs in the same patient [4,5,6]. However, also in immunocompetent subjects, this is a non-rare event that, in the literature, is estimated to vary from 5.6% to 29.2%, depending on the different sampling modes and the various geographical origins of patients [7,8,9,10,11]. Overall, a 3-year risk of up to 44% for BCC development after a previous BCC was observed [10], and many published papers confirm that subjects with prior BCC history have a 30–70% chance of having a second primary BCC within 5 years [7,8,9,10,11].

Genetic factors may contribute to the appearance of multiple BCCs in the same patient. The best-known condition is represented by the Basal Cell Nevus Syndrome (BCNS or Gorlin–Goltz syndrome) [12], an autosomal-dominant disorder primarily caused by mutations in the PATCHED-1 (PTCH1) gene, leading to the inappropriate activation of the Hedgehog signaling pathway. Furthermore, Rombo and Bazex-Duprè-Cristol syndrome are inherited conditions characterized by the presence of multiple BCCs [13]. Moreover, multiple non-syndromic superficial BCCs may occur as a distinct mendelian trait [14] and more recently, the germline mutations of the BRCA1-associated protein 1 (BAP1) have been related to this condition [15]. Exogenous risk factors, such as UV radiation, chemicals (i.e., arsenic or hydrazine) or ionizing radiation [16] play a certain role. However, the hallmarks of patients who develop multiple BCCs are not yet fully clarified.

This paper aims to analyze, through both a personal case series and a review of the most recently published papers, the characteristics of immunocompetent patients affected by multiple BCCs, with the final objective to direct them towards specific surveillance programs, increasing the number of early diagnoses and optimizing healthcare resources.

## 2. Results

### 2.1. Clinical Characteristics

Table 1 resumes the clinical and pathological characteristics of our sample, which consisted of a total of 2140 patients. Among them, 1273 (59.5%) were females and 867 (40.5%) males. The median age at first diagnosis of BCC was of about 73 years for the entire sample (range 20–101 years) and was slightly lower for women (median 71.2, range 25–101 years) than for men (median 73.8, range 20–96 years), even if this difference is not statistically significant.

The total number of BCCs excised in this patient’s series was 3222. As expected, most BCCs (60.9%) were located on the face, and the most represented subsite was the nose (26.7%). On the contrary, the anatomical site less frequently involved was the upper limbs (5%). The most frequently observed histotype was represented by a nodular BCC (64.7%, including 57 pigmented BCC); superficial and morphea-like BCCs corresponded, respectively, to 21.1% and 8.7%, and other histotypes, overall, to the remaining 5.4%.

### 2.2. Patients with Multiple BCC

From the total, 7 patients were excluded from the analysis as they were affected by BCNS syndrome, and 39 were solid organ transplantation recipients. Thus, we evaluated 2094 subjects. Overall, patients affected by multiple BCCs were 506, accounting for 24.2% of this sample. The total number of BCCs diagnosed in these cases was 1515, ranging from 2 to 11 in the same patient, as detailed in Table 2. 

Clinical characteristics of patients with multiple BCCs, divided according to the number of tumors, are summarized in Table 3, and compared with those of patients with a single BCC.

Among patients with multiple BCCs, 338 were males (66.7%) and 168 females (33.3%); this difference is statistically significant (*p* < 0.01). The percentage of male patients arose to 75.4% in the group with more than 4 BCCs. The median age of patients affected by multiple BCCs (evaluated at the time of the first diagnosis) was of 75 years (range 33–97) and was significantly higher than that of patients with a single BCC. Furthermore, we also observed an increase in age at diagnosis parallel to the number of diagnosed lesions.

In 190 patients, multiple BCCs were diagnosed synchronously. In most patients with synchronous lesions (77.6%), 2 BCCs were found. In the 17.9% of cases, there were three synchronous lesions, whereas there was very rarely four or more (3.12% and 2.08%, respectively). Moreover, 170 patients developed multiple BCCs within 1 year from their first diagnosis. The number of patients affected by metachronous lesions arising in different time intervals is shown in detail in Table 3. Interestingly, the mean interval between the appearance of the first and second BCC was 16.4 months, which decreased to 14.6 months between the second and the third, and 10.1 months between the third and the fourth tumor. For all the 1588 patients diagnosed with a single BCC, the follow-up was prolonged until the end of February 2020. None of them developed further BCC during this time interval.

One-hundred and sixteen patients affected by 2 BCCs (41.3%) showed both lesions in the same anatomical area. This percentage drops to 22.2% for patients affected by three BCCs, and to 5.9% for patients affected by four or more BCCs. Interestingly, the percentage of multiple BCCs localized to the back was higher than that of single BCCs arising in this site (23.6% vs. 15.6%), whereas, in most patients affected by a single BCC, tumors developed mainly on the head (42.8% vs. 23.6%); these differences are statistically significant.

### 2.3. Histopathological Features

Considering all the sample, the most diagnosed histotype was represented by nodular (60.5%), followed by superficial (24.8%), and infiltrative BCCs (11.6%), whereas basosquamous and pigmented subtypes accounted for 2% and 1.1%, respectively.

Table 4 summarizes the histotype of multiple BCCs in patients with two to four lesions. More than half (50.5%) of patients affected by two BCCs had both tumors of the same histological subtype. This percentage decreased to 25% for patients with three BCCs, whereas in cases of four BCCs only 12.5% of patients were affected by tumors belonging to the same histotype.

### 2.4. Risk Factors for Multiple BCCs

Risk factors for multiple BCCs are summarized in Table 5. Unfortunately, given the retrospective nature of the study, it was not possible to assess all patients for these risk factors. Data regarding skin type and smoking habits were available only in 443 patients with a single BCC and in 155 patients with multiple BCCs. These data are resumed in Table 5, but in consideration of their smallness compared to the totality of the sample, it was not possible to perform a conclusive evaluation. Data about chronic sun exposure (i.e., outdoor work or participation in outdoor activities on a daily or regular basis) were available in 1166 patients affected by a single tumor and in 406 with multiple BCCs. Even if the percentage of chronically sun-exposed patients was slightly higher in the group affected by multiple lesions (16.9% vs. 14.3%), this difference does not reach statistical significance. The same group of patients was investigated for chronic diseases with a potential immunosuppressive role; in this case, differences were non-statistically significant.

No patient reported a previous history of X-ray exposure. The retrospective nature of the stage did not allow us to investigate the possible familiarity.

In multivariate analysis, age, gender, anatomical site, and histology of the first tumor, phototype, sun exposure history, chronic diseases, and smoking were entered as variants (see Table 6). The analyses identified the male gender, an age older than 75 years at diagnosis of the first BCC, and a first BCC with a nodular histotype and/or located on the face, as prognostic indicators for the development of multiple BCCs.

## 3. Materials and Methods

### 3.1. Patient Population and Follow-Up

This study was a retrospective observation of all patients diagnosed and surgically treated for BCC from 1 January 2010 through 31 December 2015, at our Dermatological Division. The study received approval from the local Ethics Committee (CE 149/17). The follow-up of the patients included in the study was extended to February 2020 to ensure an adequate observational time span from the first diagnosis of BCC. The subsequent period was excluded from the analysis due to the impact of the COVID-19 pandemic on access to healthcare facilities

Solid-organ transplantation recipients and patients affected by the basal cell nevus syndrome (i.e., Gorlin–Gotz syndrome) were excluded from the evaluation. Patients treated with alternative treatments to surgery (imiquimod, photodynamic therapy, or other conservative treatments) were also excluded from the evaluation.

### 3.2. Statistical Analysis

Student’s *t*-test and Chi-square test were used to analyze differences on the distribution of variables (gender, age, presence of exogenous risk factors, anatomical location, and histopathological type of the tumor) between groups of patients with single or multiple BCCs.

Patients were grouped according to the number of developed BCCs. We separately considered patients with two, three, and four tumors, while, to avoid excessive fragmentation of results, those with five or more tumors were considered together as a single group. Multiple BCCs were defined as ‘synchronous’ when diagnosed within 60 days from the excision of the first lesion.

Univariate and multivariate analyses were performed to evaluate the potential role of clinical characteristics of patients and of different risk factors in developing multiple BCCs. For the multivariate analysis, we considered the following categorical data: gender (M vs. F); age (<75 vs. >75); histotype (nodular vs. others); anatomical site (face vs. others); phototype (I/II vs. III/IV); sun exposure (yes vs. no); chronical diseases (yes vs. no); smoking (yes vs. no).

## 4. Discussion

The development of multiple BCCs in the same individual is a frequent occurrence, not only among patient carriers of specific genetic abnormalities [12,13,14,15], or those who underwent chronic immunosuppressive therapies ([4,5,6], but also in non-immunosuppressed patients. Nevertheless, while risk factors in the development of multiple BCCs are well known in both syndromic patients and in carriers of solid organ transplant, or autoimmune diseases, those that characterize immunocompetent subjects are not yet fully identified.

### 4.1. Incidence of Multiple BCC in Immunocompetent Patients

There are few case studies published on this kind of patient [7,8,9,10,11,17,18,19,20,21,22], and estimating the real incidence of multiple BCCs in non-immunocompromised subjects is difficult. In our experience, the percentage of patients affected by more than one BCC is approximately 24%, in line with what is reported in different series [17,21] and also recently confirmed in a nationwide study performed in the Netherlands, which extrapolated the number of diagnoses from cancer registries evaluating more than 600,000 patients over 20 years [22]. However, this percentage was lower than that observed in other studies, where a median follow-up extended to 10 years allowed the identification of further BCCs in almost half of patients undergoing previous excision [18].

### 4.2. Timing of Onset

In our experience, multiple BCCs have been removed as synchronous in more than a third of cases (37.5%), even if the likelihood of multiple simultaneous diagnoses decreased for patients with numerous lesions (3.12% and 2.08% for 4 or more).

Moreover, another 28% of patients developed additional lesions within one year of the first diagnosis. Interestingly, the mean interval between the appearance of the first and second BCC was 16.4 months which decreased to 14.6 months between the second and the third, and further decreased to 10.1 months between the third and the fourth tumor. These data are like those also observed in a transplanted patient’s cohort, in which we proved a progressive reduction in the latency time between one neoplasm and the subsequent [23].

### 4.3. Anatomical Sites

We observed that the presence of multiple BCCs in the same anatomic area is a frequent event, even if the possibility that all lesions occur in the same site decreases with the number. Recently, Kuo et al. [18] reported, on a series of 1419 patients, a prevalence of multiple BCCs located in the head and neck region, in accordance with what has been previously reported by other authors [20]. On the contrary, in our experience, multiple BCCs developed more frequently on the back, whereas lesions located on the face or scalp were more commonly diagnosed in patients with a single tumor. Additionally, this finding agrees with the report of Bartos and Kullovà [21], who described multiple BCCs more commonly located in the trunk and upper limbs, and less frequently on the face. The concept of field cancerization [24] could justify this finding. Moreover, Zalaudek et al. [25] noticed similar clinical and dermoscopic characteristics in multiple BCCs arising in the same anatomical region on the same patient. Additionally, this “signature pattern” could be indicative of a concurrent and concomitant etiopathogenesis.

### 4.4. Histotypes

Several authors [18,26,27,28], report a significantly higher number of superficial BCCs among patients affected by multiple tumors. However, in our experience, nodular BCCs were the most frequent histotype. This discrepancy could be explained by the fact that our study was based on the analysis of medical records of patients undergoing surgery at our department: the gold standard for the treatment of nodular BCC is surgery, while many superficial BCCs could also be susceptible to conservative therapies [29,30,31]. For this reason, their actual incidence could be underestimated in our series. However, we decided not to exclude patients with histologies other than nodular from this study in order to have a more complete view of the extent of the multiple BCC phenomenon in immunocompetent patients. Furthermore, the inclusion of all histotypes has shown to have statistical significance, as demonstrated by the multivariate analysis. Based on our experience the hypothesis according to which patients with multiple BCCs are carriers of histotypes at lower risk than patients with a single BCC is not fully supportable. It is, however, interesting to note that more than half of patients with multiple BCCs develop tumors with the same histotype; this is further confirmation of a possible common etiopathogenetic origin. In our series, the presence of a nodular BCC at the first diagnosis has been shown to be an independent prognostic factor in determining the risk of multiple BCCs, as confirmed by the multivariate analysis.

Regarding the possible role of other skin cancers, Marcil and Stern [32] conducted a literature review and meta-analysis to evaluate the occurrence of subsequent NMSC according to the type of the initial NMSC, demonstrating that patients with a history of SCC had an equivalent risk of developing BCC to patients with a history of BCC. Conversely, a previous BCC does not change the risk of developing SCC.

### 4.5. Endogenous Risk Factors

Male gender and a higher age at initial BCC diagnosis are associated with metachronous BCCs [6,8,17,18,19]. A statistically significant correlation between these clinical characteristics and the development of multiple BCC has also been demonstrated in our series, in which the percentage of males and the median age at diagnosis increase further in patients with more than two BCCs. These data are also in agreement with other previous reports; a retrospective analysis performed in 2017 identified multiple BCCs in the 19% of analyzed patients, with a mean number of tumors higher in males than in females (1.5 vs. 1.2) [2]. However, some aspects remain debated: among the risk factors reported by Bartos [21], the young age at diagnosis seems to play a favoring role. It is conceivable that, in these patients, specific genetic mutations favoring the onset of BCC may be identifiable, while in older patients the most relevant role is certainly played by the cumulative action of exogenous carcinogens, including chronic UV exposure.

With regards to gender, the male sex was significantly associated, not only generically with the development of BCC, but also more specifically with the risk of multiple lesions, in our sample. This could be justified by several elements, not only genetic but also cultural elements, such as more frequent outdoor work activities, with a consequential greater exposure to the sun, scarce photoprotection habits, and less adherence to skin self-examination behaviors.

### 4.6. Mutational Pattern

Whole-exome sequencing data from sporadic BCCs found that these tumors are the most mutated of any human cancer studied thus far, with a mutational profile of BCC consistent with UV-induced mutagenesis [33].

Constitutive activation of Hedgehog (Hh) signaling is the most common primary driver in both hereditary and sporadic BCCs (reviewed in [34,35]). Germline inactivation of one copy of the Hh receptor Patched 1 (PTCH1) gene [36] and, more rarely, of PTCH2 [37], or of Suppressor of Fused (SUFU) [38], followed by the somatic loss of the second allele results in Gorlin syndrome [12] that are frequently observed in BCC patients. Furthermore, UV-induced mutations of genes encoding for Hh pathway components were identified in about 85% of sporadic BCCs [33,39,40,41,42,43,44]. Based on this evidence, the Hh pathway has become a major target for molecular therapies aimed to inhibit BBC growth, mainly through the inactivation of the Hh effector SMO (i.e., vismodegib, sonidegib, patidegib, and itraconazole) [45]. Very recently, the generation and characterization of simple BCC mouse models have unveiled key mechanisms for BCC progression, possibly related to multiple BCCs [46]. Canonical mutations that activate upstream Hh signaling are necessary, but secondary mutations that further hyperactivate downstream Hh signaling (including the amplification of Gli1/2 and upregulation of N-Myc signaling) are required in those models to escape dormancy and produce uncontrolled expansion [46].

In addition, disruption of other cancer-associated pathways may confer an increased risk for BCCs and possibly contribute to the development of multiple BCCs. Due to the critical role of UV irradiation in the pathogenesis of BCC [47], the alteration of DNA repair mechanisms has been investigated as additional driver mutations. As expected, reduced DNA repair capacity for UV–induced DNA damage is present in most BCCs and this defect is an independent risk factor for BCC [39]; furthermore, the inherited common variation in some DNA repair genes has been correlated to the development of BCCs [48,49,50]. Other mutated pathways, which may represent additional drivers, are the p53 (61% of BCCs), the N-Myc (30%), the Hippo-YAP (8%), the EGFR (4%), and the RAS (2%) pathways [33,51].

Recently, a correlation between non-melanoma skin cancer and immunosurveillance has started to be investigated. In particular, the efficiency of local immune surveillance of the cutis may limit the rate of appearance of multiple BCCs. Toward this hypothesis, mounting evidence points to an association between stemness and cancer immune evasion and resistance [52]. Recently, an integrated analysis of single-cell and bulk RNA sequencing data has identified a gene signature that may represent a robust link between cancer stemness and immunotherapy resistance for several cancers, including BCC [53].

### 4.7. Exogenous Risk Factors

Photoexposure habits may justify the different incidence of multiple BCCs and the involvement of different anatomical sites in various geographic areas [8,27,28,54]. However, although the chronic photoexposure is a major risk factor for the onset of NMSCs, the actual role of the sun in the development of multiple BCCs is still debated [8,18,55,56]. Sunburn after the age of 60 has been shown to be an independent factor significantly associated with multiple BCCs [56], and sun exposure was also confirmed to be the main risk factor for the occurrence of multiple or recurrent BCCs in a large case series recently published in Italy [57]. However, no relationship between pigment status or UV-related characteristics (easily sunburned, outdoor work, and sun protection) and the development of a second BCC has been demonstrated in other studies [19,27,28] in line with our experience. However, a limitation of our study is represented by the fact that questionnaires administered to patients considered only sun exposure related to outdoor work activity, whereas that related to recreational or outdoor activities was not investigated. Furthermore, the assessment of the extent of the photoexposure and of any previous sun burns was carried out on the basis of anamnestic data, and probably underestimated.

The literature also suggests that radiotherapy [58], especially if administered at high doses and on previously photo-damaged skin, and chemicals, such as arsenic and hydrazine (mostly used as agricultural chemicals or as a corrosion inhibitor in heating systems) [59] can play a putative role in etiopathogenesis of multiple BCCs. In our case series, we investigate a possible correlation with smoking or chronic diseases that could potentially down-regulate the immune system activity, but we have not proven any significant relationship. Interestingly, a recent study suggested that coffee consumption reduced the risk of a second BCC [60]. This observation is supported by experimental evidence showing that, in mice, oral and topical administration of caffeine inhibits UVB-induced carcinogenesis and selectively increase apoptosis in NMSCs [61].

## 5. Conclusions

In conclusion, the development of multiple BCCs in immunocompetent patients is a frequent, often underestimated event. Therefore, the identification of the higher risk subjects is essential for the development of specific health educational and surveillance programs, also with a view to resources optimization. In the literature [40,51], it has been proposed that the strongest predictor of further BCCs is the presence of more than one BCC at initial diagnosis. In our experience, a patient’s gender and age (i.e., male and elderly), and tumor histotype and localization (i.e., nodular and face), also depict the phenotype at higher risk for development of multiple lesions. In these subjects, the cumulative exposure to UV radiation represents a possible additional risk factor, while the role of somatic mutations is still to be definitively clarified.

Our experience, in agreement with the literature data [40,51], confirms that most subsequent BCCs develop within 1 year from the first diagnosis; however, the non-inconsistent percentage of multiple BCCs diagnosed after 5 and even 10 years [18] makes the long-term continuation of this follow-up mandatory. In fact, only the identification of new BCCs as early as possible allow the use of conservative therapeutic modalities, and reduce the need for invasive or complex surgical procedures (i.e., Mohs surgery), resulting in less discomfort for the patient and savings in health spending.

Among the possible therapeutic alternatives, based on the high frequency of mutations, systemic treatment with hedgehog pathway inhibitors has also been proposed in non-syndromic patients affected by multiple BCCs. A subgroup of patients with these characteristics was included both in the pivotal phase 2 registration study ERIVANCE BCC [62], and in the randomized phase 2 trial MIKIE, that assessed the safety and activity of two long-term intermittent vismodegib dosing regimens [63]. Moreover, in 2020, Verkouteren et al. [64] conducted a retrospective cohort study, also including two high-frequency BCC patients. However, the effectiveness of the drug in this specific patient’s subset needs to be still clarified.

## Figures and Tables

**Table 1:** Clinical and pathological characteristics of patients affected by BCC.

No. of pts	2140
Age * (median, range)	73.8 (20–101)
Females	1273 (59.5%)
Males	867 (40.5%)
Head	60.9%
*Nose*	*517 (26.7%)*
*Ear*	*223 (11.5%)*
*Scalp*	*197 (10.2%)*
*Eyelid*	*179 (9.2%)*
*Lips*	*56 (2.9%)*
*Others*	*764 (39.5%)*
Back	620 (19.2%)
Chest	329 (10.2%)
Legs	174 (5.4%)
Arms	163 (5%)
Histotype	
Nodular	2086 (64.7%)
Superficial	679 (21.1%)
Morphea-like	282 (8.7%)
Basosquamous	61 (1.9%)
Cystic	58 (1.8%)
Other	56 (1.7%)

* At first diagnosis of BCC.

**Table 2:** Patients affected by multiple BCC.

No. of Tumors	Patients
2	281 (55.5%)
3	108 (21.3%)
4	48 (9.5%)
5	28 (5.5%)
6	18 (3.6%)
7	10 (2%)
8	3 (0.6%)
9	6 (1.2%)
10	3 (0.6%)
11	1 (0.2%)

**Table 3:** Clinical characteristics of patients with single or multiple BCCs.

	Single BCC	Multiple BCCs	2 BCCs	3 BCCs	4 BCCs	>4 BCCs
No. of pts	1588	506	281	108	48	69
Age * (median, range)	71 (20–101)	75 (33–97)	75 (33–97)	75 (40–90)	77 (43–93)	74 (39–92)
*Gender*						
Female	734 (46.2%)	168 (33.2%)	106 (37.7%)	30 (27.7%)	15 (31.25%)	17 (24.3%)
Male	854 (53.8%)	338 (66.8%)	175 (62.3%)	78 (72.3%)	33 (68.75%)	52 (75.7)
*p* < 0.01						
*Anatomical site °*						
Head	680 (42.8%)	470 (31%)	28 (9.9%)	4 (3.7%)	1 (2%)	0
*Nose*	*333 (48.9%)*	*172 (36.6%)*	*17 (60.7%)*	*1 (25%)*	*0*	*0*
*Ear*	*101 (14.8%)*	*118 (25.1%)*	*3 (10.7%)*	*2 (59%)*	*0*	*0*
*Scalp*	*92 (13.5%)*	*103 (21.9%)*	*2 (7.1%)*	*1 (25%)*	*1*	*0*
*Eyelid*	*121 (17.7%)*	*55 (11.7%)*	*6 (21.4%)*	*0*	*0*	*0*
*Lips*	*33 (4.8%)*	*22 (4.6%)*	*0*	*0*	*0*	*0*
Neck	376 (23.6%)	389 (25.6%)	29 (10.3%)	4 (3.7%)	0	0
Back	248 (15.6%)	327 (21.6%)	20 (7.11%)	8 (7.4%)	2 (4%)	2 (2.9%)
Chest	146 (9.2%)	157 (10.3%)	9 (3.2%)	1 (0.9%)	0	0
Legs	73 (4.6%)	90 (5.9%)	1 (0.003%)	3 (2.7%)	1 (2%)	0
Arms	65 (4%)	83 (5.4%)	1(0.003%)	0	0	0
*Timing of BCCs*						
Synchronous	/	190 (37.5%)	149 (53%)	34 (31.5%)	6 (12.5%)	1 (1.4%)
Less than 1 year ^§^	/	170 (33.5%)	68 (24.2%)	36 (33.3%)	18 (37.5%)	48 (69.5%)
1 year < 3 ^§^	/	118 (23.3%)	53 (18.9%)	31 (28.7%)	16 (33.3%)	18 (26%)
3 years < 5 ^§^	/	28 (5.5%)	11 (3.9%)	7 (6.5%)	8 (16.6%)	2 (2.8%)

* At first BCC diagnosis; ° number of patients with 2 or more BCCs occurring in the same anatomical site; ^§^ from the first BCC diagnosis.

**Table 4:** Histopathological features of patients with single or multiple BCCs.

	Single BCC	2 BCCs	3 BCCs	4 BCCs	>4 BCCs
No. of pts No. of lesions	1588 1588	281 562	108 324	48 192	69 438
*Histotype (1st)*					
Nodular	1084 (68.26%)	274	118	46	81
Superficial	249 (15.7%)	83	49	32	48
Infiltrative	216 (13.6%)	56	27	9	17
Basosquamous	39 (2.4%)	7	3	0	0
Histotype (2nd) *					
Nodular	/	87	57	38	77
Superficial	/	34	25	20	31
Infiltrative	/	21	12	9	11
Basosquamous	/	0	4	2	2
Histotype (3rd) *					
Nodular	/	/	21	22	57
Superficial	/	/	4	5	27
Infiltrative	/	/	2	3	4
Basosquamous	/	/	0	0	3
Histotype (4th) *					
Nodular	/	/	/	2	28
Superficial	/	/	/	1	17
Infiltrative	/	/	/	2	4
Basosquamous	/	/	/	1	1

* All synchronous lesions were considered together.

**Table 5:** Risk factors for single or multiple BCCs.

	Single BCC	Multiple BCCs	2 BCCs	3 BCCs	4 BCCs	>4 BCCs
Phototype						
I/II	239 (53.9%)	87 (56.1%)	51	16	9	11
III	184 (41.5%)	66 (42.5%)	33	18	5	10
IV	20 (4.6%)	2 (1.4%)	2	0	0	0
Sun exposure						
Yes	167 (14.3%)	69 (16.9%)	38	14	7	10
No	999 (85.7%)	337 (83.1%)	188	76	29	44
Chronic diseases						
Yes	509 (43.6%)	198 (48.7%)	108	45	19	26
No	657 (56.4%)	208 (51.3%)	118	45	17	28
Smoking						
Yes	172 (38.8%)	58 (37.4%)	34	12	6	6
No	271 (61.2%)	97 (62.6%)	52	22	8	15

**Table 6:** Multivariate analysis of risk factors for the development of multiple BCCs.

	HR	95% CI	*p*
Gender (M vs. F)	1.39	1.141–1.703	0.001
Age (>75, vs. <75)	1.01	1.008–1.023	<0.001
Histology (nodular, vs. others)	0.85	0.732–0.988	0.034
Anatomical site (face, vs. others)	1.54	1.335–1.795	<0.001
Phototype (I, II vs. III, IV)	0.977	0.707.1.349	0.88
Sun exposure (yes, vs. no)	0.98	0.725–1.346	0.988
Chronical diseases (yes, vs. no)	1.18	0.969–1.451	0.099
Smoking (yes, vs. no)	0.97	0.696–1.364	0.879

## Data Availability

The data presented in this study is available on request from the corresponding author.
